# Cellular Immune Response to Parasitization in *Drosophila* Requires the EBF Orthologue Collier

**DOI:** 10.1371/journal.pbio.0020196

**Published:** 2004-08-17

**Authors:** Michèle Crozatier, Jean-Michel Ubeda, Alain Vincent, Marie Meister

**Affiliations:** **1**Centre de Biologie du Développement, Centre National de la Recherche Scientifique and Université Paul SabatierToulouse, France; **2**Institut de Biologie Moléculaire et CellulaireCentre National de la Recherche Scientifique, StrasbourgFrance

## Abstract

*Drosophila* immune response involves three types of hemocytes (‘blood cells’). One cell type, the lamellocyte, is induced to differentiate only under particular conditions, such as parasitization by wasps. Here, we have investigated the mechanisms underlying the specification of lamellocytes. We first show that *collier (col),* the *Drosophila* orthologue of the vertebrate gene encoding early B-cell factor (EBF), is expressed very early during ontogeny of the lymph gland, the larval hematopoietic organ. In this organ, Col expression prefigures a specific posterior region recently proposed to act as a signalling centre, the posterior signalling centre (PSC). The complete lack of lamellocytes in parasitized *col* mutant larvae revealed the critical requirement for Col activity in specification of this cell type. In wild-type larvae, Col expression remains restricted to the PSC following parasitization, despite the massive production of lamellocytes. We therefore propose that Col endows PSC cells with the capacity to relay an instructive signal that orients hematopoietic precursors towards the lamellocyte fate in response to parasitization. Considered together with the role of EBF in lymphopoiesis, these findings suggest new parallels in cellular immunity between *Drosophila* and vertebrates. Further investigations on Col/EBF expression and function in other phyla should provide fresh insight into the evolutionary origin of lymphoid cells.

## Abstract 2

The lamellocyte is induced to differentiate under conditions such as parasitization by wasps and is shown in this study to require collier, the orthologue of the vertebrate early B-cell factor

## Introduction

Hematopoiesis in *Drosophila* shares several features with the analogous process in vertebrates. A first population of embryonic hemocyte precursors (prohemocytes) is specified from the head mesoderm very early during embryogenesis. At the end of larval stages and the onset of metamorphosis, a second population of hemocytes is released from a specialised hematopoietic organ, the larval lymph gland (Rizki and Rizki 1984; Tepass et al. 1994; Campos-Ortega and Hartenstein 1997; Evans et al. 2003; Holz et al. 2003). Both populations give rise to plasmatocytes, which are dedicated phagocytes, and crystal cells, which are responsible for melanisation of pathogens. Lymph glands contain precursors of a third type of hemocyte that is not generated in embryos, the lamellocyte. Lamellocytes are large, adhesive cells devoted to the encapsulation of foreign bodies too large to be phagocytosed; these cells differentiate only in response to specific conditions, such as parasitization of larvae by Hymenoptera (Lanot et al. 2001; Sorrentino et al. 2002). Striking similarities with vertebrate hematopoiesis were revealed when it was shown that Serpent (Srp), a GATA factor, and Lozenge (Lz), a transcription factor related to Runx1/AML1, are required for the development of hemocytes and of crystal cells, respectively (Rehorn et al. 1996; Lebestky et al. 2000; Orkin 2000). However, except for the observation that gain-of-function mutations in the Janus kinase Hopscotch and in the Toll receptor lead to constitutive production of lamellocytes (Harrison et al. 1995; Luo et al. 1995; Qiu et al. 1998), the mechanisms and factors underlying the specification of this cell type remain unknown (Evans et al. 2003; Meister 2004).

During our search for genes involved in specification of lamellocytes, we observed that *collier (col)* is expressed in the lymph glands at the end of embryogenesis (Kambris et al. 2002). The gene *col* encodes the *Drosophila* orthologue of mammalian early B-cell factor (EBF) (Hagman et al. 1993; Crozatier et al. 1996), a key factor controlling B-cell lymphopoiesis in mice (Lin and Grosschedl 1995; Maier and Hagman 2002). We show here that Col activity is required for specification of the lamellocyte lineage in *Drosophila.* On the basis of Col expression and *col* mutant phenotypes, we propose that this factor confers an instructive function on a discrete subpopulation of cells in the *Drosophila* definitive hematopoietic organ.

## Results/Discussion

### Col Expression Identifies Lymph Gland Precursors in Early Embryos

**Figure 1:**
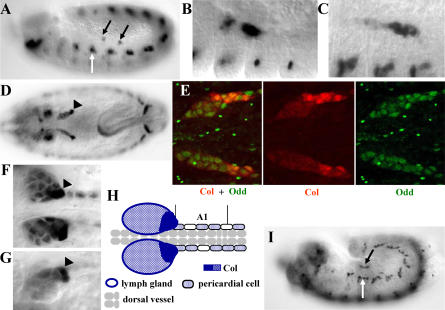
Col Expression during Lymph Gland Ontogeny (A) Col expression in lymph gland precursors is first observed in two separate clusters of cells (black arrows) in the dorsal-most mesoderm of thoracic segments T2 and T3 at stage 11 (stages according to Campos-Ortega and Hartenstein [1997]). Col expression in the head region is ectodermal (parasegment 0) and related to its function in head segmentation (Crozatier et al. 1999). (B and C) The clusters of Col-expressing cells get closer between stage 12 and early stage 13 (B) before coalescing (C). (D and E) Col expression becomes progressively restricted to the posterior-most cells of the forming lymph glands (arrowhead) during stage 14, as shown by the partial overlap between Odd-skipped (Odd) and Col expression. (F and G) Enlarged view of lymph glands after completion of embryogenesis, stage 16. Col expression marks the prospective PSC (Lebestky et al. 2003) in a dorsal-posterior position (arrowheads). (H) Schematic representation of Col expression in the lymph glands and pericardial cells in stage 16 embryos. (I) A *srp^6G^* mutant embryo arrested at stage 13. Col is expressed in the presumptive lymph gland primordium (black arrow), although it is not possible to distinguish between high and low levels of expression. All embryos are oriented anterior to the left. (A–C), (G), and (I) are lateral views; (D–F) are dorsal views. (B), (C), and (E–G) are higher magnifications of the dorsal thoracic region. White arrows in (A) and (I) indicate Col expression in a developing dorsal muscle (Crozatier and Vincent 1999).

We first observed that Col is expressed in *Drosophila* lymph glands at the end of embryogenesis (Figure 1). In the absence of a specific molecular marker, the embryonic anlage of lymph glands has been mapped to the thoracic lateral mesoderm by lineage analysis of transplanted cells (Holz et al. 2003). By histochemical staining, we observed that Col is expressed in two discrete clusters of cells in the dorsal mesoderm of thoracic segments T2 and T3, starting at the germ-band extension, when lymph gland hemocyte precursors become specified (stage 11; Figure 1A) (Holz et al. 2003). These clusters of Col-expressing cells grow closer during germ-band retraction before coalescing to form the paired lobes of the lymph glands (early stage 13; Figure 1B and 1C). Double staining for Col and Odd-skipped, a lymph gland marker expressed from that stage onward (Ward and Skeath 2000), confirmed that Col-expressing cells are lymph gland precursors (Figure 1E). Thereafter, only three to five cells located at the posterior tip of each lobe maintain high levels of Col expression, although low levels are still detected in the other cells of the lymph glands and in some pericardial cells (Figure 1D, 1F, and 1G). Col expression thus identifies a few cells of the thoracic dorsal mesoderm as the lymph gland primordium and distinguishes a specific posterior region of this hematopoietic organ (Figure 1H). The embryonic hematopoietic primordium has been defined as the cephalic domain of Srp expression at the blastoderm stage (Rehorn et al. 1996; Lebestky et al. 2000). Srp is not detected, however, in lymph gland precursors prior to stage 12 (Berkeley Drosophila Genome Project gene expression report [http://www.fruitfly.org/cgi-bin/ex/insitu.pl]**;** Lebestky et al. 2003). Consistent with this result, larval hematopoietic progenitors expressing Col are observed in *srp^6G^* (an amorphic allele; Rehorn et al. 1996) mutant embryos (Figure 1I), indicating that the specification of the embryonic and larval lymph gland progenitors may involve different processes.

### Lamellocyte Differentiation in Response to Parasitization Requires Col Activity

**Figure 2:**
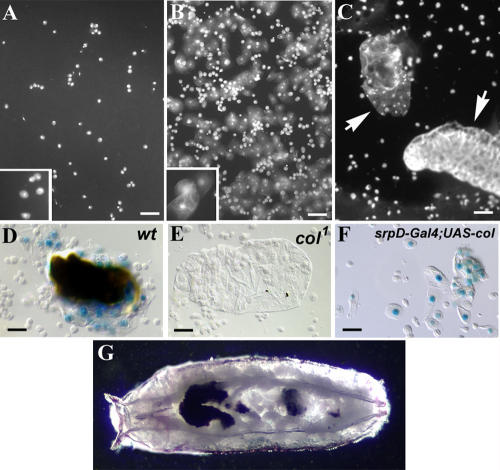
*col* Requirement for Lamellocyte Differentiation (A–C) 4′,6-diamidino-2-phenylindole (DAPI) staining of hemocytes from *wt* (A and B) and from *col^1^* (C) third instar larvae. (A) Uninfected larva; (B) and (C) infected larvae. Plasmatocytes (inset in [A]) are always present, whereas lamellocytes (inset in [B]) are detected in the hemolymph of *wt* (B) but not *col^1^* (C) larvae 48 h after infestation by *L. boulardi.* In *col^1^* mutants, the wasp eggs are not encapsulated (white arrows) and develop into larvae (bottom right organism in [C]). (D–F) Lamellocytes expressing the P-*lacZ* marker *l(3)06949* (Braun et al. 1997) surround the wasp eggs in *wt* larvae (D), are completely absent in infected *col^1^* mutant larvae (E), and differentiate in the absence of wasp infection following enforced Col expression in hematopoietic cells (*srpD-Gal4/UAS-col* larvae) (F). (G) *srpD-Gal4/UAS-col* pupa showing the presence of melanotic tumors. Bars: 50 μm.

Expression of Col in the embryonic lymph gland prompted us to investigate its possible function during larval hematopoiesis. Loss-of-function mutations of *col* (e.g., *col^1^*) are lethal at the late embryonic stage (Crozatier et al. 1999), but lymph glands form normally, indicating that Col activity is not required for formation of the organ per se. Rescue of the embryonic lethality by expressing the *col* cDNA under the control of a truncated *col* promoter that is active in the head ectoderm but not in the lymph glands (Crozatier and Vincent 1999) thus allowed us to analyse hematopoiesis in *col^1^* larvae. The presence of plasmatocytes and crystal cells in the circulation of these mutants indicated that *col* is not required for specification of either of these lineages (Table 1). We then tested the competence of *col^1^* larvae to respond to wasp *(Leptopilina boulardi)* parasitization by producing lamellocytes. This dedicated cellular response is maximal in wild-type *(wt)* larvae 48 h after wasp egg-laying (Figure 2A and 2B) (Lanot et al. 2001). No circulating lamellocytes were detected in the hemolymph of parasitized *col^1^* larvae; as a consequence, the wasp eggs were not encapsulated and they developed into parasitic larvae (Figure 2C). That this phenotype completely lacked lamellocytes was confirmed by using a lamellocyte marker, *misshapen-lacZ,* provided by the enhancer trap line *l(3)06949* (Braun et al. 1997). Whereas in *wt* larvae, numerous *lacZ*-positive cells could be seen adhering to and surrounding wasp eggs, no such cells were detected in *col^1^* larvae (Figure 2D and 2E). To ascertain that the absence of lamellocytes was the consequence solely of the *col* mutation, we tested *col^1^* in transheterozygous combinations with two other *col* loss-of-function alleles and over the deficiency *Df(2R)AN293* (Crozatier and Vincent 1999). In no case did we observe lamellocyte differentiation (we tested 10–20 larvae for each genotype) in response to parasitization by *L. boulardi,* thereby confirming the critical requirement for Col activity in rendering hematopoietic precursors competent to differentiate into lamellocytes. Although gain-of-function mutations that lead to constitutive activation of either the Janus kinase or the Toll signalling pathways result in hematopoietic defects, including differentiation of lamellocytes in the absence of infestation (Harrison et al. 1995; Luo et al. 1995; Qiu et al. 1998), *col^1^* is, to our knowledge, the first identified loss-of-function mutation that abolishes lamellocyte production upon parasitization.

**Table 1:**
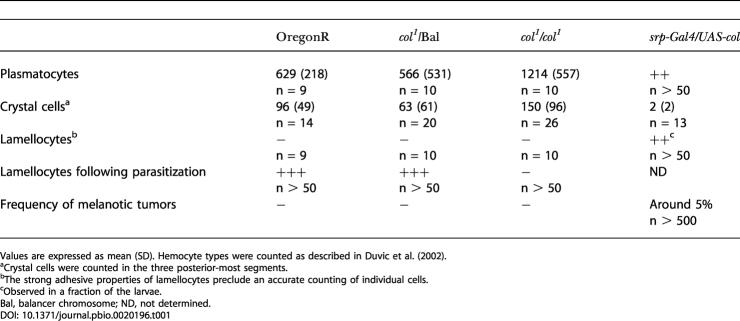
Circulating Hemocytes in Third Instar Larvae

Values are expressed as mean (SD). Hemocyte types were counted as described in Duvic et al. (2002)

^a^Crystal cells were counted in the three posterior-most segments

^b^The strong adhesive properties of lamellocytes preclude an accurate counting of individual cells

^c^Observed in a fraction of the larvae

Bal, balancer chromosome; ND, not determined

### Enforced Col Expression Triggers Lamellocyte Differentiation in the Absence of Immune Challenge

We then asked whether forced expression of Col in hematopoietic cells could induce lamellocyte differentiation in the absence of infestation. Because the *e33C-Gal4* line, which drives expression in lymph glands (Harrison et al. 1995) but also epidermis and some other tissues, was lethal in combination with *UAS-col,* we designed a new Gal4 driver. The driver *srpD-Gal4* contains distal elements of the *srp* gene promoter and drives expression of a *UAS* reporter gene in prohemocytes and hemocytes (see below) (Waltzer et al. 2003), with a low level of expression in pericardial cells and the fat body (data not shown). Although embryonic-lethal at 25 °C, the *srpD-Gal4/UAS-col* combination was viable when embryos were allowed to develop to the second larval instar at 18 °C before shifting to 25 °C. Examination of hemolymph samples from late third instar larvae expressing Col under the control of the *srpD-Gal4* driver revealed the presence, in a fraction of the larvae, of numerous lamellocytes identified on the basis of both cell morphology and expression of *misshapen-lacZ* (Figure 2F; Table 1). Around 5% of all larvae developed melanotic tumors (Figure 2G), which have been previously observed in other genetic contexts that lead to overproduction of lamellocytes (Hou and Perrimon 1997). This phenomenon is considered to be a consequence of an autoimmune reaction in which hemocytes encapsulate self-tissue (Sparrow 1978). Thus, we conclude that enforced *col* expression in hematopoietic cells can induce differentiation of lamellocytes in the absence of immune challenge. We also observed a concomitant drop in the number of circulating crystal cells (Table 1), consistent with the hypothesis that lamellocytes and larval crystal cells could differentiate from a common precursor (Evans et al. 2003). No production of lamellocytes was observed, however, when *col* expression was targeted to already specified crystal cells or plasmatocytes by using the *lz-Gal4* (Lebestky et al. 2000) and *hml-Gal4* drivers (Goto et al. 2003), respectively. This indicates that lamellocytes differentiate only when *col* expression is forced in yet-uncommitted progenitors.

### Col-Expressing Cells Play an Instructive Role

**Figure 3:**
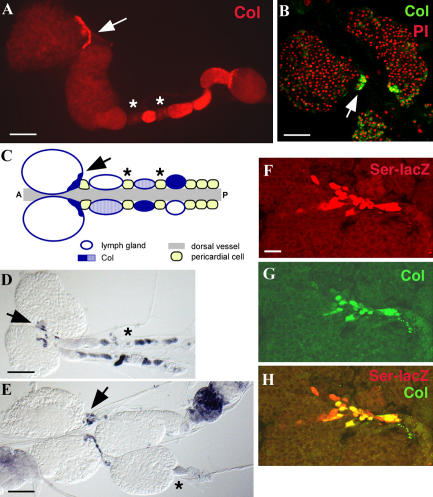
Col Expression in Lymph Glands of Third Instar Larvae (A and B) Col is expressed in the primary lobes, in a posterior cluster of cells (arrow), and in a variable number of secondary lobes. Low expression is also detected in some pericardial cells (asterisks), the significance of which remains unknown. PI, propidium iodide. (C) Schematic representation of the lymph glands and Col expression in late third instar larvae. (D and E) Col expression 24 h (D) and 48 h (E) after wasp infection; despite strong cell proliferation, including in secondary lobes, Col expression remains unchanged (black arrow). (F–H) Overlap between Ser-lacZ (Bachmann and Knust 1998) and Col expression in PSC cells; note a few scattered Ser-expressing cells that do not stain for Col. Bars: 50 μm (A, B, D, and E) ; 10 μm (F–H).

At the end of larval stages, the lymph gland is composed of four to six paired lobes. The two anterior (primary) lobes that formed in the embryo (Figure 1) contain prohemocytes, plasmatocytes, and crystal cells, whereas the posterior (secondary) lobes, which form during the third larval instar, contain predominantly prohemocytes, suggesting that they correspond to a more immature stage of development (Shrestha and Gateff 1982; Lanot et al. 2001). Col expression in the anterior primary lobes was found to be restricted to a posterior cluster of about 30–40 posterior cells (Figure 3A–3C). Consistent with, on average, three to four cell divisions between embryo hatching and the third larval instar—as observed both in circulating hemocytes and imaginal tissues (Schubiger and Palka 1987; Qiu et al. 1998)—these cells are likely to represent the entire progeny of the three to five cells that strongly express Col in the late embryo (see Figure 1E and 1F). They remain clustered at the posterior end of the primary lobes throughout larval development. Col is expressed in a variable number of cells in secondary lobes (Figure 3A and 3C) but is never observed in circulating hemocytes. Despite the dramatic burst of lamellocyte production that occurs in lymph glands when larvae are parasitized (see Figure 2) (Lanot et al. 2001; Sorrentino et al. 2002), the number and posterior clustering of Col-expressing cells were unchanged (Figure 3D and 3E). This indicates that the small group of Col-expressing cells are not likely to be the direct precursors of lamellocytes, but rather that they play an instructive role in orienting hematopoietic precursors present in the lymph glands towards the lamellocyte lineage.

**Figure 4:**
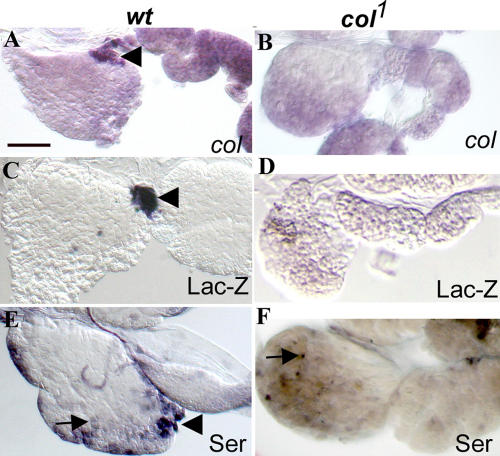
PSC-Specific Gene Expression Is Dependent upon Col Activity PSC-specific expression of *col*, Ser-lacZ, and Ser (arrowhead in [A], [C], and [E]) is lost in *col^1^* mutant larvae (B, D, and F); only Ser expression in scattered cells is maintained (arrow in [E] and [F]). Bar: 50 μm.

Col expression in a posterior cluster of cells of the primary lobes is reminiscent of that of Serrate (Ser), a Notch ligand (Lebestky et al. 2003). The Ser/Notch pathway has recently been shown to be essential for crystal cell development (Duvic et al. 2002; Lebestky et al. 2003). Analysis of clones of *Ser* mutant cells in the larval lymph glands further indicated that Ser-expressing cells are responsible for activation of Lz expression in surrounding cells and their commitment to a crystal cell fate (Lebestky et al. 2003). Together with the Ser expression pattern, this observation led the authors to propose that the posterior cluster of Ser-expressing cells could act as a signalling centre, which they termed the posterior signalling centre (PSC). Through double-labelling experiments, we confirmed the overlap between Col and Ser expression (as visualised by Ser-LacZ [Bachmann and Knust 1998]) in the posterior cells of the primary lobe (Figure 3F–3H). However, Ser, but not Col, is expressed in scattered cells throughout the primary lymph gland lobes in addition to the PSC (Figure 4) (Lebestky et al. 2003).

### PSC-Specific Gene Expression Is Dependent upon Col Activity

Because Col expression and function suggested that the PSC was playing an instructive role in orienting other lymph gland cells towards the lamellocyte fate, we asked whether Col was necessary for the PSC to form properly. We looked at *col* and *Ser* expression in *col^1^* mutant lymph glands, using in situ hybridisation for *col* because Col antibodies do not recognise the Col^1^ protein (Crozatier and Vincent 1999). In *wt* larvae, consistent with the results of immunostaining, *col* transcripts were restricted to the PSC (Figure 4A). In contrast, we could not detect *col* expression in *col^1^* mutant lymph glands (Figure 4B). Furthermore, expression both of Ser-lacZ and Ser in the PSC (Figure 4C and 4E) was also abolished (Figure 4D and 4F), indicating that proper specification of PSC identity is dependent upon Col activity. Although Ser expression was lost from the PSC region, it was still observed in scattered cells in the primary lobe (Figure 4E and 4F, arrows), suggesting that Ser-lacZ expression reflected the presence of a PSC-specific transcriptional enhancer without reproducing the entire Ser expression pattern.

### Evidence for a Bipotential Crystal Cell/Lamellocyte Precursor

**Figure 5:**
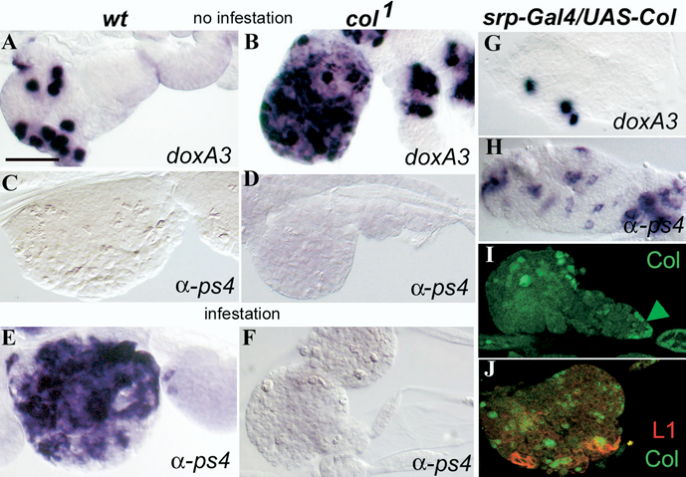
Col-Expressing Cells Play an Instructive Role in Lamellocyte Production Expression of the crystal cell marker *doxA3* (Waltzer et al. 2003) (A, B, and G); of the lamellocyte markers *α-ps4* (M. Meister, unpublished data) (C–F and H) and L1 (Asha et al. 2003) (J); and of Col (I and J); in *wt* (A, C, and E), *col* loss-of-function mutant (B, D, and F), and *srp-Gal4/UAS-col* (G–J) larvae. In (E) and (F), larvae were taken 48 h after infestation. An increased number of *doxA3*-positive cells (B) parallels the absence of lamellocyte differentiation (F) in *col^1^* mutant lymph glands. Conversely, lamellocyte differentiation and a reduced number of *doxA3*-positive cells are observed upon enforced Col expression (G and H). Double staining for Col and L1 shows that Col-expressing cells and differentiating lamellocytes do not overlap in the lymph gland. (I) shows ectopic Col expression compared to expression in the PSC (arrowhead; not visible in [J]). Antibody and in situ probes are indicated on each panel. In all panels, larvae are oriented with the head to the left: a single primary lobe is shown, with sometimes a few secondary lobes. Bar: 50 μm.

Ser signalling through the Notch signalling pathway is critical for the specification of crystal cell precursors (Duvic et al. 2002; Lebestky et al. 2003). However, numerous crystal cells differentiate in *col* mutant lymph glands, including in secondary lobes, despite the loss of Ser expression in the PSC (see Figures 4E, 4F, 5A, and 5B). These data, together with the clonal analysis of Lebestky et al. (2003), lead us to conclude that crystal cell development is triggered by signalling from the scattered Ser-expressing lymph gland cells, rather than from the PSC itself. In contrast, no differentiating lamellocytes could be detected in *col* mutant lymph glands, even under conditions of wasp infestation that induced massive lamellocyte differentiation in *wt* glands (Figure 5C–5F), confirming the key role of the PSC in this process.

We then looked at the production of crystal cells and lamellocytes in lymph glands with enforced Col expression (*srpD-Gal4/UAS-col*; Figure 5G–5J). Very few crystal cells and numerous lamellocytes were observed, consistent with the circulating hemocyte picture (Figure 5G and 5H). The *srpD-Gal4*-driven Col expression in the lymph gland is not uniform. Some cells express high levels when compared to the PSC, whereas many others show no detectable expression. A similar pattern was also observed in combination with UAS-lacZ (Figure 5I; data not shown). Double-labelling experiments showed that the lymph gland cells induced to differentiate into lamellocytes surround but do not overlap with the Col-expressing cells (Figure 5J), confirming the instructive role of Col-expressing cells. In all genotypes that we tested, we found equally large numbers of plasmatocytes in the lymph glands (data not shown), which indicates that this cell type is not affected by *col* loss-of-function and gain-of-function mutations. Altogether, the absence of lamellocytes after parasitization that is associated with the increase in the number of crystal cells in *col* mutant lymph glands, and the opposite situation in *srpD-Gal4/UAS-col* lymph glands (Figure 5; Table 1), support the existence of bipotential crystal cell/lamellocyte precursors.

### A Model for Induction of Lamellocytes in Response to Parasitization

In summary, our data show that (i) Col expression defines a specific group of cells within the lymph glands; (ii) lamellocyte differentiation, which is an exclusive feature of lymph gland hematopoiesis, depends upon Col activity; and (iii) the massive production of lamellocytes that follows parasitization does not involve changes in Col expression. We thus propose a two-step signalling model for induction of lamellocytes in response to wasp egg-laying (Figure 5A). According to this scheme, Col endows PSC cells with the competence to respond to a primary signal emitted by plasmatocytes as these permanent immune supervisors form a first layer around the parasite egg (Russo et al. 1996). Subsequently, PSC cells send a secondary signal that orients prohemocytes towards the lamellocyte fate. The production of lamellocytes upon enforced *col* expression suggests that the need for the primary signal to activate the secondary signal can be bypassed in overexpression experiments. Although several aspects of this model remain to be translated into molecular terms, it certainly sheds a new light on the genetic control of hemocyte lineages in *Drosophila.*

### Concluding Remarks

**Figure 6:**
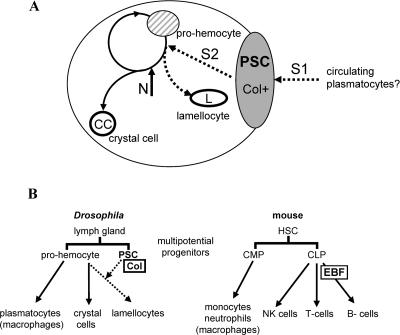
A Model for Lamellocyte Specification (A) A model for the induction of lamellocyte differentiation in the *Drosophila* lymph glands in response to wasp parasitization. Col enables PSC cells to respond to a primary signal (S1) that is likely emitted by plasmatocytes upon their encounter with a parasite (Russo et al. 1996; Meister 2004). As a result, the PSC cells send a secondary signal (S2) that causes prohemocytes to develop into lamellocytes. Notch (N) signalling instructs a fraction of prohemocytes to become crystal cells (Duvic et al. 2002; Lebestky et al. 2003). The circular arrow indicates that increased proliferation leading to increased numbers of crystal cells and lamellocytes follows parasitization (Sorrentino et al. 2002). (B) Schematic view of hematopoiesis in *Drosophila* and mouse*.* Left: Lymph gland cells contain two types of hematopoietic cells, PSC cells and uncommitted precursors. These precursors can give rise to either plasmatocytes or crystal cells. Crystal cell precursors can also give rise to lamellocytes upon receiving a signal from the PSC cells expressing Col (dotted arrows); this signalling is itself dependent upon a communication between circulating plasmatocytes and the PSC (A). Right: In mice, hematopoietic stem cells (HSC) give rise to common myeloid precursors (CMP) and common lymphoid precursors (CLP) (adapted from Orkin [2000] and Schebesta et al. [2002]). Signalling between CMP- and CLP-derived cells is an essential component of adaptive immunity. Col and EBF functions, in *Drosophila* and vertebrate hematopoiesis, respectively, suggest an ancestral role in their conferring on a subset of hematopoietic cells the ability to respond to signals from circulating immune supervisors (generically designated here as macrophages) and to provide a secondary line of defence against specific intruders.

B- and T-lymphocytes mediate adaptive immunity, a phylogenetically recent component of the immune system as it is found only in gnathostomes (Kimbrell and Beutler 2001; Mayer et al. 2002). How adaptive immunity emerged during evolution, and was built on top of the innate immune system by which it is controlled and assisted, remains a fascinating question. The requirement for Col function in the *Drosophila* cellular immune response, and EBF function in B-cell development in vertebrates, suggests that Col/EBF function was co-opted early during the evolution of cellular immunity. A puzzling question remains, however, of how the cell-autonomous function of EBF in B-cell development, and the non–cell-autonomous function of Col in lamellocyte development, could relate to an ancestral Col/EBF function. We would like to propose that the ancestral expression of Col/EBF in a subset of hematopoietic cells conferred on these cells the ability to respond to signals from circulating immune supervisors (generically designated as macrophages in Figure 6) and provide a secondary line of defence against specific intruders. This cell-specific property in turn laid the ground for the emergence of the vertebrate lymphoid cells on one side and the *Drosophila* PSC on the other. Although admittedly highly speculative, this proposal takes into account the following considerations. B-cell development represents the default fate of lymphoid progenitors (Schebesta et al. 2002; Warren and Rothenberg 2003). Although specification of B-cells critically depends on EBF (and the basic helix-loop-helix protein E2A), commitment depends on another gene, *Pax5.* The *Pax5^−/−^* pro–B-cells retain the ability to generate a whole range of both ‘innate’ myeloid and lymphoid cells (Nutt et al. 1999; Rolink et al. 1999; Mikkola et al. 2002). Thus, the ontogeny of the B-cell lineage from preexisting myeloid cell types has occurred through several steps, one key event being the co-opting of *Pax5,* acting downstream of EBF, for which there is no known counterpart in *Drosophila* hematopoiesis. Second, the co-opting of Col activity for lamellocyte differentiation in larval hematopoiesis most likely came on top of a preexisting hematopoietic system, such as that operating in *Drosophila* embryos (Evans et al. 2003; Meister 2004). Further investigation of Col/EBF functions in intermediate phyla should provide more insight into the diversity of myeloid lineages and ontogeny of the lymphoid lineages during evolution.

## Materials and Methods

### 

#### Fly stocks and hemocyte counting

Unless otherwise stated, all fly stocks were maintained at 25 °C on standard medium, and genotypes were verified with marked balancer chromosomes. For wasp infection, second instar larvae were submitted to egg-laying by L. boulardi for 2–4 h, then allowed to develop at the appropriate temperature and analysed 24 or 48 h later. Hemocyte observation and counting, and *lacZ* staining of lamellocytes, were as previously described (Braun et al. 1997; Duvic et al. 2002).

#### Transgenic constructs and flies

The *srpD-Gal4* transgene: A distal promoter fragment, between 8.8 and 6 kb upstream of the *srp* transcription start site and a 340-bp fragment overlapping this site were amplified by PCR using 5′-GCTAGCGACGCGTGATGCAACTTAATCAA-3′ and 5′-CTGCAGTTTATGAATGGAAGACGCGGACG-3′ primers, and 5′-CTGCAGACGGCCAAGTCCAACAACAACAA-3′ and 5′-GGATCCCTGTTGCTGCTGTAACTGTTGAT-3′ primers, respectively, then fused before subcloning upstream of the *Gal4* coding sequence in a pCaSpeR vector. Transgenic lines were obtained by standard procedures. Because they are embryonic-lethal at 25 °C, the *srpD-Gal4/UAS-col* animals were kept at 18 °C before shifting to 25 °C at the second larval instar.

#### Immunostaining and in situ hybridisation.

Immunostaining and in situ hybridisation of larval lymph glands and embryos were performed as in Crozatier and Vincent (1999) using rabbit anti-Col (1:250), rat anti-Ser (gift from K. Irvine; 1:500), mouse anti-β-galactosidase (Promega, Madison, Wisconsin, United States; 1:1000), mouse lamellocyte-specific L1 (gift from I. Ando; 1:10), and guinea-pig anti-Odd-skipped (gift from J. B. Skeath; 1:100). Peroxidase and Alexa Fluor 546 or 488 labelled secondary antibodies (Molecular Probes, Eugene, Oregon, United States) were used at a 1:500 dilution. In some cases, lymph glands were incubated for 30 min at 37 °C in a propidium iodide solution in the presence of RNase. Mounting in Vectashield medium (Vector Laboratories, Burlingame, California, United States) preceded observation by confocal microscopy (Zeiss LSM 510 [Zeiss, Oberkochen, Germany] and Leica SP2 [Leica, Wetzlar, Germany]). Single-stranded digoxigenin-labelled RNA probes were synthesised from corresponding cDNAs cloned in pGEM (Promega).

## Abbreviations

Col: Collier
EBF: early B-cell factor
Lz: Lozenge
PSC: posterior signalling centre
Ser: Serrate
Srp: Serpent
*wt*: wild-type
